# Fixing geriatric ankle fractures: fibular nail versus locking plate in a prospective multicenter study

**DOI:** 10.1007/s00068-026-03108-5

**Published:** 2026-02-23

**Authors:** Felix C. Kohler, Zoe Berfelde, Philipp Schenk, Wolfram Weschenfelder, Britt Wildemann, Philipp Kobbe, Thomas Mendel, Bernhard W. Ullrich

**Affiliations:** 1 Department of Trauma, Hand and Reconstructive Surgery and Orthopedics, Jena University Hospital, Friedrich Schiller University Jena, 07747 Jena, Germany; 2 Innovationhub for Musculoskeletal Surgery Halle, BG Klinikum Bergmannstrost Halle gGmbH, Merseburger Straße 52, 06110 Halle (Saale), Germany; 3 Department of Trauma and Reconstructive Surgery, BG Klinikum Bergmannstrost Halle gGmbH, 06112 Halle, Germany; 4 Department of Trauma and Reconstructive Surgery, University Hospital Halle, Martin Luther University Halle/Wittenberg, Halle, Germany

**Keywords:** Ankle fracture, Geriatrics, Fibular nail, Locking plate, Early weight-bearing, Complications

## Abstract

**Purpose:**

Elderly patients with unstable ankle fractures face a high risk of wound and implant-related complications after open reduction and internal fixation (ORIF). Less invasive intramedullary fibular nail (FN) fixation may reduce soft-tissue trauma and enable earlier mobilization.

**Methods:**

In this prospective multicenter trial with pseudorandomized allocation and protocol-permitted crossover, 55 geriatric multimorbid patients (Charlson Comorbidity Index ≥ 4; mean age FN 79 ± 8 vs. ORIF 74 ± 8 years; *p* = 0.053) were treated with FN (*n* = 39) or ORIF (*n* = 16). Primary outcomes were operative time, fluoroscopy time, hospital stay, weight-bearing at discharge, complications, and functional scores (AOFAS, OMAS, Weber) at six weeks and twelve months.

**Results:**

FN required more fluoroscopy time (*p* = 0.011), while operative duration and hospital stay were comparable (*p* = 0.176, *p* = 0.520). Full weight-bearing at discharge was more frequent after FN (62% vs. 0%; *p* < 0.001). At six weeks, FN patients achieved higher functional scores (AOFAS *p* = 0.041; OMAS *p* = 0.027), but at twelve months no differences remained (AOFAS *p* = 0.404; OMAS *p* = 0.288; Weber *p* = 0.585). Radiographic malalignment was more common after FN (46% vs. 13%; *p* = 0.031). Distal screw loosening at twelve months occurred more often after FN (80% vs. 11%; *p* = 0.005) but was mostly asymptomatic.

**Conclusion:**

Earlier mobilization observed after FN in our study primarily reflects the permissive postoperative weight-bearing protocol rather than an inherent biomechanical advantage of the implant. At twelve months, functional outcomes were equivalent, underscoring that protocol-driven early loading, not implant design, explains early differences.

## Background

Ankle fractures are among the most common skeletal injuries, affecting both younger active individuals and older adults [1–3]. In geriatric patients, these injuries are frequently associated with osteoporosis and multimorbidity. Complex patterns, such as ankle dislocations or syndesmotic injuries, typically require surgical stabilization to restore joint congruence [4]. In this population, the standard treatment is open reduction and internal fixation (ORIF) using locking plates and screws [5].

However, ORIF in elderly patients carries a substantial risk of postoperative complications, particularly wound complications (7–13%) and implant-related infections [5–8]. Zaghloul et al. identified age, comorbidities, and smoking as major risk factors, reporting an overall complication rate of 22%, with 11% requiring reoperation [7]. Frail patients are also at risk from immobilization, underscoring the importance of early weight-bearing and mobilization. Therefore, minimally invasive surgical approaches help to reduce soft tissue trauma and support these aims in this population. Intramedullary fibular nail (FN) fixation has emerged as a promising technique, especially in patients with significant comorbidities [9–11].

Recent randomized controlled trials and meta-analyses have demonstrated comparable functional and radiographic outcomes between fibular nailing and plate fixation, with lower soft-tissue complication rates but similar union and reoperation rates for both techniques. The most recent RCT-based meta-analysis by *Cook et al.* (2025) confirmed the overall equivalence of both procedures, underscoring that implant selection should primarily consider patient factors, comorbidity, and the potential for early mobilization [12].

FN shows fewer wound complications due to its minimally invasive approach. Anatomical reduction may be more challenging, but functional outcomes remain comparable to ORIF [13–16]. Biomechanical studies have shown heterogeneous results [17, 18]. The biomechanical advantages of nail osteosynthesis include higher torsional stiffness compared to locking plate osteosynthesis [19]. The potential for immediate full weight bearing makes FN particularly attractive for geriatric patients [5, 20, 21].

This prospective, multicenter study with pseudorandomized allocation specifically addresses geriatric, multimorbid patients with ankle fractures, comparing fibular nailing to standard ORIF in terms of both early mobilization and long-term outcomes.

## Methods

### Study reporting

**Fig. 1:**
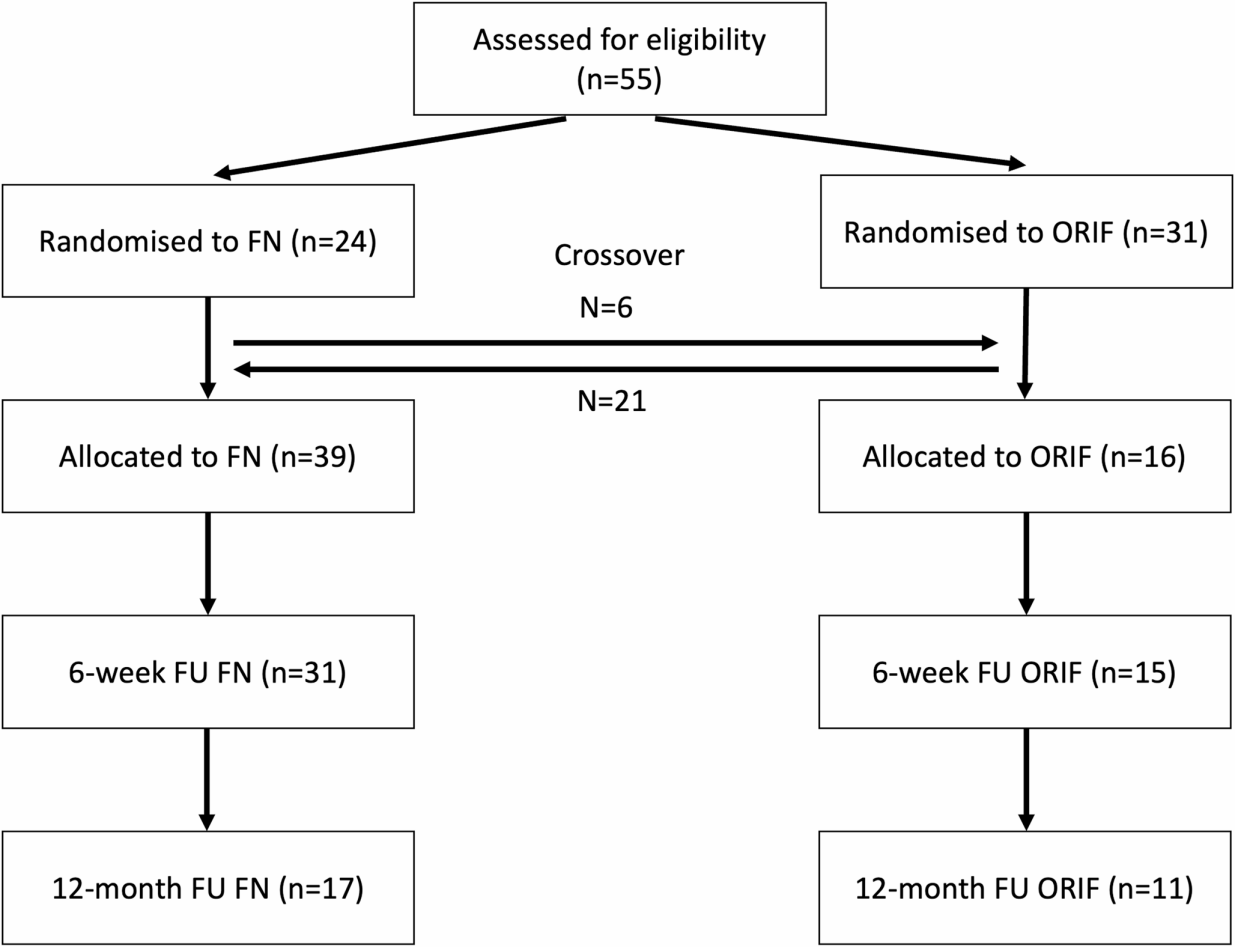
Study flow diagram

The reporting of patient flow and study conduct follows the Consort extension for nonrandomized and pragmatic trials. A Consort-style flow diagram outlining enrollment, allocation, crossover events, follow-up and analysis is provided in Fig. 1.

### Enrollment

Between February 2020 and December 2021, all patients presenting with an ankle fracture at the two participating German level I trauma centers were screened for eligibility.

### Allocation

Treatment allocation followed a predefined pseudorandomized alternating sequence (FN / ORIF) applied at each enrolling center. This ensured balanced group distribution but did not constitute full randomization.

### Crossover criteria

Crossover from the allocated intervention was allowed under predefined, surgeon-determined criteria:

1. Failure to achieve or maintain adequate closed reduction.
2. Poor local soft-tissue conditions making the allocated procedure unsuitable.
3. Intraoperative concern that the planned implant would not provide safe fixation.

All crossover events were documented prospectively, and the analysis was performed in an as-treated fashion.

### Follow-up

Patients were evaluated perioperatively, at 6 weeks and at 12 months. At each time point, clinical, radiological, and functional outcomes were collected using predefined case report forms.

Follow-up completion rates were:

- 6 weeks: FN 31/39 (79%), ORIF 15/16 (94%).
- 12 months: FN 17/39 (44%), ORIF 11/16 (69%).

### Analysis

All outcomes were analyzed on an as-treated basis, consistent with the study design. Data collection included clinical examination, radiographic assessment, surgical details, perioperative metrics, and functional outcome measures.

Of 55 patients assessed for eligibility, 24 were allocated to fibular nailing (FN) and 31 to open reduction and internal fixation (ORIF). Crossover between treatment arms occurred according to predefined clinical criteria, resulting in 39 patients treated with FN and 16 with ORIF in the as-treated cohort. Follow-up was available at 6 weeks for 31 FN and 15 ORIF patients, and at 12 months for 17 FN and 11 ORIF patients.

### Implants used

The following implants were used depending on the surgical procedure: for fibular nailing, exclusively the VITUS-Fi Fibula Nail System (Axomed / Marquardt Medizintechnik, Spaichingen, Germany); for ORIF, the VariAx Distal Fibula Locking Plate System (Stryker, Kalamazoo, MI, USA) or CP™ Distal Fibula Plate System (DePuy Synthes, Zuchwil, Switzerland).

### Patient population and ethical approval

Ethical approval was obtained from the local institutional review boards (40/19 state medical association Saxony-Anhalt), and written informed consent was provided by all patients.

### Inclusion and exclusion criteria

Inclusion criteria:

- Signed informed consent.
- CCI ≥ 4.
- Ankle fracture type ≥ AO-44B1 / Lauge-Hansen stage 2.

The AO/OTA classification system categorizes ankle fractures as type 44, with subtype B injuries representing trans-syndesmotic fractures and subtypes B1–B3 indicating increasing complexity [22]. According to the Lauge-Hansen classification, supination–external rotation (SER) injuries progress through four stages, with stages 3–4 indicating increasing medial and syndesmotic instability [23].

Exclusion criteria:

- Proximal AO-44C2 or AO-44C3 fractures.
- Pathological fractures.

The Charlson Comorbidity Index (CCI) accounts for age and comorbidities and is widely used to estimate perioperative risk. A threshold of ≥ 4 was chosen because the age of 80 and more by itself defines a CCI of 4, so this value reflects a state of frailty at least like an octogenarian [24].

### Data collection

Demographic, clinical, radiological, and functional data were collected preoperatively, perioperatively, and at 6 weeks and 12 months. Functional outcomes were assessed using the American Orthopaedic Foot & Ankle Society score (AOFAS [25]), the Olerud-Molander Ankle Score (OMAS [26], ), and the Weber score [27].

The X-Ray subscore of Webers score was used to analyze quality of reduction. Additional parameters included the incidence of complications, the need for revision surgery, the use of assistive devices for mobilization (e.g., crutches, walkers, or orthoses), and the patients’ weight-bearing status (full, partial/assisted, or non-weight-bearing). Postoperative weight-bearing protocols were defined by the treating surgeon. Although immediate full weight-bearing is recommended by the manufacturer after fibular nailing, it was only exceptionally permitted following ORIF, resulting in a systematic difference between groups regarding postoperative loading.

### Subgroup classification

Fracture stability was classified according to the Lauge-Hansen classification. Only supination–external rotation (SER) and pronation-type injuries were included, as no Weber A-type fractures underwent surgical treatment. Fracture stability was classified according to Lauge-Hansen and Weber. Weber B type fractures without medial involvement (SER stage ≤ 3) were considered as stable, while Weber B fractures with medial involvement (SER stage 4) and all Weber C fractures were defined as unstable.

### Statistical analysis

Categorical variables were analyzed using Fisher’s exact test, and continuous variables with general linear models. GLM for repeated measures (GLMrm) was applied for the functional scores. Subgroup analyses were conducted for stable versus unstable fractures. Differences between patients with stable and unstable fracture types in the functional outcome (AOFAS, OMAS, Weber) at six and twelve months were analyzed using GLM for repeated measures. Interaction effects between the fixed factor (FN or ORIF) and time points identified as statistically significant in the GLMs are reported with their corresponding p-values. Non-significant interactions are not reported to improve clarity and readability. Unless otherwise specified, interval-scaled data are presented as means with standard deviations, while categorical variables are summarized using frequencies and corresponding percentages. Lower and upper limits of the 95% confidence interval (95% CI) are given in addition to the standard deviation for the AOFAS, OMAS and Weber score. All analyses were performed with IBM SPSS Statistics 27 (IBM Corp., Armonk, NY, USA), with *p* < 0.05 considered statistically significant.

## Results

### Study population

A total of 55 patients (16 male, 39 female), FN *N* = 39 and ORIF *N* = 16, were included with follow-up data available for 46 patients at six weeks (FN *N* = 31, ORIF *N* = 15, follow-up: 83.6%), and 28 patients at twelve months (FN *N* = 17, ORIF *N* = 11, follow-up: 50.9%), respectively. Follow-up was completed in 79% (31/39) of patients in the FN group and 94% (15/16) in the ORIF group at 6 weeks, and in 44% (17/39) versus 69% (11/16) at 12 months, respectively. This corresponds to dropout rates of 21% at 6 weeks and 49% at 12 months. Twelve-month dropout differed between groups (FN 56% vs. ORIF 31%), representing an important limitation**.** The distribution between the two treatment groups showed no statistically significant differences across the observation time points (*p* = 0.357).

Age tended to be higher in the FN group (79 ± 8 vs. 74 ± 8 years, *p* = 0.053). The CCI did not significantly differ between both groups, with a mean of 5.5 ± 1.6 (FN) and 5.7 ± 1.6 (ORIF, *p* = 0.628). Fracture patterns also differed not significantly between groups (*p* = 0.068, Table 1). Operative duration and hospital stay did not differ between both groups (*p* = 0.176), whereas fluoroscopy time was significantly longer for FN (*p* = 0.010, Table 1). Detailed baseline characteristics and operative parameters are summarized in Table 1.

**Table 1:** Baseline characteristics, operative parameters, and hospital stay in FN and ORIF groups

	FN	ORIF	p
Cohort			
total	39	16	
f	28	13	
m	11	3	0.522
6 wks	31	15	
12 mo	17	11	
Age (y)	79±8	74±8	0.053
CCI	5.5±1.6	5.7±1.6	0.628
Operative time (min)	75±34	88±23	0.176
Fluoroscopy time (s)	59±46	25±22	0.010
Hospital stay (d)	13±11	11±5	0.520
Fracture type			0.068
AO 44B1	4	0	
AO 44B2	11	3	
AO 44B3	15	8	
AO 44C1	9	2	
AO 44C2	0	3	

### Functional outcome

At discharge, 62% of FN patients and none of the ORIF patients were allowed full weight-bearing, reflecting the predefined postoperative protocols. Because weight-bearing status was protocol-determined rather than randomized, no inferential statistics were applied. At six weeks, 87% of both groups required assistive devices (FN *n* = 27, ORIF *n* = 13; *p* = 1.000). By twelve months, this declined to 41% in the FN group and 45% in the ORIF group (*p* = 1.000). Among the 25 patients who attended both follow-ups, 48% (*n* = 12) used assistive devices throughout the study.

At six weeks, FN patients achieved significantly higher AOFAS and OMAS scores (*p* = 0.041, *p* = 0.027) compared to the ORIF group. After twelve months no significant differences between both groups in the functional outcomes, including the Weber score, could be found. Both groups showed improvement over time in all functional outcome scores (Table 2), except for the FN group in the AOFAS score. Although the score at twelve months was higher than at six weeks, the difference was not statistically significant (*p* = 0.124). Detailed functional results are presented in Table 2.

**Table 2:** Functional outcome scores (AOFAS, OMAS, Weber score) at 6 weeks and 12 months in FN and ORIF groups, given as mean±standard deviation and the lower and upper limits of 95% confidence interval in brackets. “p within”: intragroup comparison between time points; “p between”: intergroup comparison for each time point

		6 wks	12 mo	*P* within
AOFAS	FN	75 ± 17 (67–84)	83 ± 20 (73–93)	0.124
	ORIF	61 ± 16 **(51–72)**	77 ± 18 **(65–89)**	0.015
	P between	0.041	0.404	
OMAS	FN	51 ± 25 **(40–63)**	72 ± 21 **(61–83)**	< 0.001
	ORIF	30 ± 19 **(16–45)**	63 ± 22 **(49–76)**	< 0.001
	P between	0.027	0.288	
Weber score	FN	1.6 ± 0.9 **(1.1–2.0)**	0.91 ± 0.8 **(0.5–1.3)**	0.001
	ORIF	2.0 ± 0.9 **(1.5–2.6)**	1.1 ± 0.9 **(0.6–1.6)**	< 0.001
	P between	0.209	0.585	

### Complications

**Fig. 2:**
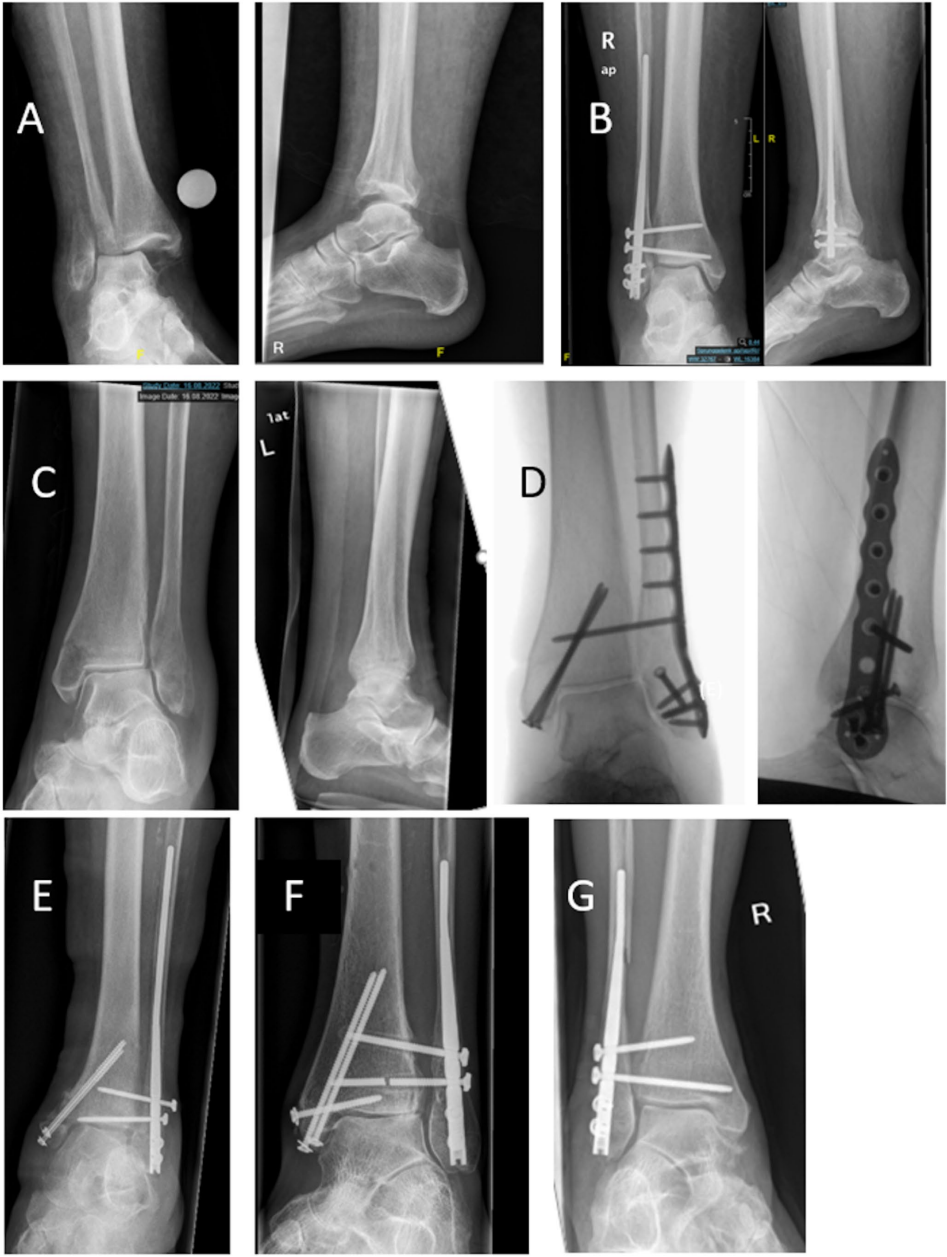
(**A**) Preoperative radiographs prior to fibular nail osteosynthesis., (**B**) Postoperative result after fibular nail fixation with interfragmentary screws. (**C**) Preoperative radiographs prior to subsequent plate osteosynthesis. (**D**) Postoperative result after open reduction and internal fixation (ORIF) with locking plate and screws. (**E**) Complication example after fibular nailing with screw migration/loosening. (**F**) Complication example with breakage of the syndesmotic screw after fibular nail fixation. (**G**) Complication example with diaphyseal burst (“diaphyseal blowout”) following nail insertion

Intraoperative events occurred in 7 FN (18%) and zero ORIF patients (*p* = 0.090). Events in the FN group comprised loss of reduction requiring intraoperative revision (*n* = 3), distal interlocking/screw malposition with immediate correction (*n* = 2), iatrogenic cortical fissure without consequence (*n* = 1, Fig. 2G), and transient hardware jamming resolved intraoperatively (*n* = 1).

Postoperative complications did not differ significantly between groups at discharge, six weeks, or 12 months (Table 3). Reoperations were required in 3 patients (8%) in the FN group before discharge versus none in the ORIF group (*p* = 0.548). After six weeks, reoperations were needed in 6 FN patients (19%) and 3 ORIF (20%, *p* = 1.000) patients. At twelve months, 1 FN patient (6%) and 3 ORIF patients (27%, *p* = 1.000) required reoperations. Cumulatively, 10 of 39 FN patients (26%) and 6 of 16 ORIF patients (38%) underwent at least one reoperation during follow-up (accounting for overlap across time points). The most frequent indications were loss of reduction or implant-related malposition, prominent or migrated distal screws, and superficial dehiscence requiring revision. No deep infection occurred.

At discharge, radiographic abnormalities were more frequent after treatment with FN (46%) than with ORIF (13%, *p* = 0.031), predominantly including residual fibular shortening/rotation, medial clear space widening, or syndesmotic malreduction. At 6 weeks, radiographic abnormalities were present in 9 of 29 FN patients (31%) and in 5 of 14 ORIF patients (36%, *p* = 1.000). Radiologically fracture union was observed in 83% of FN patients and 71% of ORIF patients (*p* = 0.442). At 6 weeks, implant migration or screw loosening occurred in 9 of 29 FN patients (31%) and in 3 of 14 ORIF patients (21%, *p* = 0.720). At 12 months, radiographic abnormalities persisted in 6 of 10 FN (60%) and in 3 of 9 ORIF patients (33%, *p* = 0.370). Radiographically fracture union was achieved in all cases. At this follow-up, distal screw loosening was observed more often in the FN group (80% vs. 11%; *p* = 0.005, Fig. 2E). In most cases this represented a radiographic finding without clinical symptoms.

**Table 3:** Complication rates at discharge, 6 weeks, and 12 months in FN and ORIF groups

complications	discharge		p	6 wks		p	12 mo		p
	FN	ORIF		FN	ORIF		FN	ORIF	
	n	n		n	n		n	n	
general	6	1	0.660	3	0	0.541	2	0	0.505
Soft tissue-associated	6	3	0.710	8	6	0.495	3	3	0.653
Bone-related	1	1	0.501	3	0	0.541	0	0	-
Implant-associated	1	0	1.000	2	1	1.000	1	0	1.000

### Correlation analysis

No significant correlation was found between quality of fracture reduction and AOFAS (six weeks: *p* = 0.318; twelve months: *p* = 0.954) or OMAS (six weeks: *p* = 0.328; twelve months: *p* = 0.545). A significant moderate correlation with the Weber score was observed at six weeks (*r* = 0.511, *p* = 0.001). This correlation was no longer present at twelve months (*p* = 0.141).

### Subgroup analysis: stable vs. unstable fractures

No significant postoperative differences in weight-bearing between patients with stable and patients with unstable fracture was found (*p* = 0.236). Patients with unstable fractures showed a significant AOFAS improvement from six weeks to twelve months (mean increase 13 ± 17, **95%CI 5 and 22**; *p* = 0.004), whereas no significant change was observed in the stable group (**95%CI 12 and 18**, *p* = 0.716). Between-group differences were not significant at either time point (six weeks: *p* = 0.396; twelve months: *p* = 0.659).

For the OMAS, both groups improved significantly over time. OMAS increased in stable fractures from 50 ± 31 (**95%CI: 30 and 70**) at six weeks to 69 ± 25 (**95%CI: 51 and 86**) at twelve months (*p* = 0.023) and for unstable fractures from 41 ± 23 (**95%CI: 30 and 52**) to 68 ± 21 (**95%CI: 58 and 78**, *p* < 0.001). No significant between-group differences were observed at both observations (six weeks: *p* = 0.416; twelve months: *p* = 0.961).

The Weber score improved significantly in unstable fractures (1.9 ± 0.8, **95%CI 1.5 and 2.3** to 1.0 ± 0.8, **95%CI 0.6 and 1.3**; *p* < 0.001) but not in stable fractures (1.4 ± 1.2, **95%CI 0.6 and 2.1** to 1.0 ± 1.0, **95%CI 0.4 and 1.7**; *p* = 0.250), with no significant differences between groups at both observations (six weeks: *p* = 0.193; twelve months: *p* = 0.817).

Intraoperative and postoperative complication rates did not differ significantly between stable and unstable fractures. A detailed description is provided in Table 4. Revision surgery occurred at similar rates in both groups (discharge: 3 FN patients, 8%; ORIF 0 patients; six weeks: 6 FN patients, 19% vs. 3 ORIF patients, 20%; twelve months: one FN patient, 6% vs. 3 ORIF patients, 27%). Detailed results for these subgroups are summarized in Table 4.

**Table 4:** Complication rates at discharge, 6 weeks, and 12 months in stable versus unstable fracture subgroups

complications	discharge		p	6 wks		p	12 mo		P
	stable	unstable		stable	unstable		stable	unstable	
	n	n		n	n		n	n	
general	2	5	1.000	0	3	0.557	1	1	0.444
Soft tissue-associated	3	6	0.678	2	12	0.294	3	3	0.144
Bone-related	1	1	0.448	1	2	1.000	0	0	-
Implant-associated	0	1	1.000	2	7	1.000	0	1	1.000

## Discussion

This prospective multicenter trial with pseudorandomized allocation and protocol-permitted crossover compared fibular intramedullary nailing (FN) and open reduction and internal fixation (ORIF) in geriatric ankle fractures with significant comorbidity (CCI ≥ 4). Given the risks of extensive surgical exposure in frail patients, less invasive strategies such as FN are increasingly considered. This study provides comparative outcome data on function, radiology, and complications.

FN required nearly twice the fluoroscopy time compared to ORIF, likely reflecting the technical demands of closed reduction and the learning curve of the procedure [28]. However, fluoroscopy time is influenced by factors such as surgeon experience, device settings, and learning curve effects, and should therefore be interpreted with caution. Mean operative time did not differ significantly, though FN showed greater variability, consistent with the early adoption of minimally invasive techniques [28].

Despite earlier full weight-bearing in the FN group, hospital stay did not differ from ORIF patients, likely reflecting institutional factors.

 The systematically different weight-bearing protocols between groups did not occur arbitrarily but reflect established clinical practice and manufacturer recommendations. The fibular nail used in this study is explicitly cleared by the manufacturer for immediate full weight-bearing based on its intramedullary load-sharing design. In addition, multiple studies have demonstrated that early full weight-bearing after stable fixation of geriatric ankle fractures does not increase complication rates and is associated with faster recovery of mobility and independence [29–31]. In contrast, postoperative partial weight-bearing remains common practice after plate fixation because many surgeons consider ORIF constructs, especially in osteoporotic bone, more vulnerable to displacement under early loading [5].

These considerations explain the divergent protocols in our cohort. Importantly, it cannot be ruled out that the higher early complication rates observed after FN were influenced by immediate full weight-bearing, although the present study was not powered to isolate this effect. Therefore, postoperative loading strategy represents a relevant confounder for both clinical outcomes and complication patterns and must be taken into account when interpreting early results.

Earlier full weight-bearing (62% vs. 0% after ORIF) translated into superior six-week functional scores, with significantly higher AOFAS and OMAS values in the FN group compared to ORIF. By twelve weeks, however, functional outcomes converged and no significant differences between groups remained. This is consistent with prior reports linking early loading to faster recovery and fewer systemic complications in elderly patients [32–35]. However, evidence also indicates that full weight-bearing can be achieved after ORIF with comparable benefits [31, 36]. While FN provides sufficient stability for early loading [21], neither FN nor plating fully restores native ankle stability in highly unstable fractures [19]. By twelve months, functional outcomes (OMAS, AOFAS, Weber) converged between groups. This pattern, also reported in RCTs and systematic reviews, indicates that FN mainly accelerates early recovery rather than altering long-term results [13, 37, 38]. However, previous studies have largely focused on functional outcomes alone, often in mixed or younger populations, and were mostly retrospective or single center. Our prospective, multicenter study specifically addresses elderly multimorbid patients, integrating functional recovery, weight-bearing status, and complication profiles. This provides novel evidence on the clinical trade-offs of FN in a high-risk geriatric cohort.

Radiographic union was achieved in all patients at twelve months, consistent with previous studies [16, 21]. FN was associated with more malreductions, likely related to closed reduction, all of which resolved without functional impact. Screw loosening was markedly more frequent after FN (80% vs. 11%), a complication described in previous studies but not at the high frequency observed in our cohort [39, 40]. Although distal screw loosening was substantially more common after FN, it was largely a radiographic phenomenon without functional sequelae in our cohort, underscoring the need for routine imaging follow-up rather than reflexive reoperation in asymptomatic patients. An additional, previously undiscussed factor regarding the higher complication rates in the FN group may be related to their earlier mobilization compared to the ORIF patients. While early full weight-bearing is generally associated with improved short-term function and reduced immobilization-related complications in elderly patients [9, 41], it may also increase the risk of mechanical problems such as screw loosening or loss of reduction, particularly in cases with poor bone quality or suboptimal implant positioning [28, 40]. In our study, the FN patients were routinely permitted earlier weight-bearing, which may partly explain the higher rate of radiographic abnormalities and reoperations observed in this group. This highlights the need for careful patient selection and meticulous surgical technique when adopting immediate weight-bearing protocols after fibular nailing.

No correlation was found between anatomical alignment and AOFAS or OMAS at six weeks or twelve months, suggesting that minor malreductions may be functionally compensated in elderly patients. A moderate correlation with the Weber score was present at six weeks but not sustained at twelve months. These findings support a conservative approach toward isolated radiographic abnormalities without clinical symptoms. A limitation is that functional scores were only assessed at follow-up visits, not immediately postoperatively.

Unlike prior meta-analyses and cohort studies reporting fewer complications with FN, especially wound problems [16, 21, 32, 42], our trial found no difference in overall complication or revision rates compared with ORIF. Our findings are consistent with the RCT-based meta-analysis by *Cook et al.* (2025), which reported no significant differences in functional outcomes, infection, or reoperation rates between fibular nailing and plating, but a trend toward fewer wound complications and shorter operative times with the nail technique [12]. Together with previous evidence, this supports the view that both implants achieve similar long-term results, while fibular nailing may offer specific advantages in soft-tissue preservation and early rehabilitation in frail geriatric patients. FN patients showed more radiographic complications and hardware-related revisions, including screw loosening and secondary dislocations. This underscores that FN remains prone to complications, particularly during early adoption or with suboptimal case selection, and the differing postoperative protocols with more frequent immediate full weight-bearing after FN may also have contributed to the higher rate of implant-related problems in our cohort.

Patients with unstable fractures showed a stronger trend toward functional improvement in the Weber score, as the interaction effect narrowly missed the threshold for statistical significance despite comparable initial weight-bearing status. This suggests that instability should not be viewed as a negative prognostic factor, but rather as an indicator for operative stabilization and structured rehabilitation [43, 44]. Subgroup analysis revealed no differences in complications or revision rates between stable and unstable fractures, suggesting that standardized surgical care can equalize risk. Treatment choice between FN and ORIF should be guided primarily by patient factors rather than fracture classification alone [43]. Unstable fractures generally require surgical fixation to enable early mobilization, whereas stable fractures may be managed less invasively given comparable long-term outcomes [45]. Classification remains valuable for planning and anticipating risks, particularly concerning medial and syndesmotic integrity.

FN may be particularly suitable for elderly patients with compromised soft tissue, limited compliance with partial weight-bearing, or high perioperative risk. Its main advantages are early functional recovery and reduced soft-tissue trauma [10, 16]. However, FN should be reserved for carefully selected patients, performed by experienced surgeons, and followed by close radiographic monitoring.

### Strengths and limitations

Strengths of this study include its pseudorandomized, multicenter design and the specific focus on high-risk geriatric patients. Several limitations must also be acknowledged. The high dropout rate at 12 months, particularly in the FN group (56% vs. 31% in the ORIF group), reduces the precision of long-term comparisons and may introduce attrition bias. Postoperative loading protocols were not standardized and differed systematically between groups, representing a major confounder that directly affects early functional outcomes. Allocation followed a pseudorandomized alternating sequence with surgeon-driven crossover, which introduces potential selection bias because treating surgeons could override the assigned treatment based on clinical or intraoperative judgment. All analyses were performed in an as-treated manner rather than intention-to-treat, meaning treatment effects may have been influenced by crossover patterns. Additional limitations include the moderate sample size, incomplete follow-up due to Covid-19–related restrictions, variability in surgical technique, and the absence of stratification for fracture severity. Nevertheless, the pragmatic design reflects real-world geriatric trauma care and enhances the external validity of the findings.

## Conclusion

In elderly patients with unstable ankle fractures, FN and ORIF achieve comparable long-term outcomes. The earlier mobilization observed after FN in our cohort was primarily driven by the systematically different postoperative loading protocol, not by an inherent biomechanical advantage of the implant. Consequently, the superior six-week functional outcomes must be interpreted as protocol-dependent. FN was associated with higher rates of early malreduction and screw-related abnormalities, highlighting the importance of meticulous technique, careful intraoperative reduction control, and structured postoperative follow-up. When appropriately selected and performed by experienced surgeons, FN remains a viable option for multimorbid elderly patients, particularly those who are unlikely to comply with prolonged partial weight-bearing.

## Data Availability

The datasets used and/or analyzed during the current study are available from the corresponding author on reasonable request.
